# Beyond BACI: Offsetting carcass numbers with flight intensity to improve risk assessments of bird collisions with power lines

**DOI:** 10.1002/ece3.8291

**Published:** 2021-11-10

**Authors:** Moritz Mercker, Klaus Jödicke

**Affiliations:** ^1^ Bionum GmbH –Consultants in Biological Statistics Hamburg Germany; ^2^ Institute of Applied Mathematics (IAM) Heidelberg University Heidelberg Germany; ^3^ Biologen im Arbeitsverbund (B.i.A.) Bordesholm Germany

**Keywords:** BACI, bird collision, bird flight, bird strike, collision rate, experimental design, field design, power line, statistical method

## Abstract

1. The continuing global expansion of electricity networks increases the risk of bird collisions with power lines. Several field studies have demonstrated that this risk can be reduced by marking lines with flight diverters. A before‐after control‐impact (BACI) design is currently the suggested approach for evaluating the effectiveness of these diverters and is generally assumed to give unbiased results.
2. Using systematic flight survey data, we demonstrate that the assumptions underlying the BACI approach are frequently violated, leading to biased effectiveness estimates. We present an alternative field and statistical design in which the number of bird strike victims is directly related to bird flight intensity (“fusion design”), instead of estimating it indirectly using a control site. The presented design is validated based on simulations.
3. We demonstrate that the presented method is unbiased and shows an approximately 3‐fold higher statistical power compared with BACI, even under ideal/unbiased data conditions, with similar field‐experimental effort. Moreover, this approach can provide a direct analysis of bird reactions/collisions, estimation of collision rates, and the possibility of conducting the required fieldwork within a single season.
4. Our presented method can be used to standardize and improve future studies on diverter effectiveness, for example, by supporting the acquisition of a more detailed picture of species‐, diverter type‐, and habitat‐specific estimates.

## Graphical abstract

We compare and extend methods to analyze bird collision frequency at power lines. We present a new field and statistical approach (“fusion approach”) and demonstrate that this new method is unbiased and shows an approximately 3‐fold higher statistical power compared with previous methods.

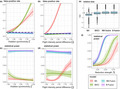

## 1 INTRODUCTION

Globally increasing energy demands in conjunction with new climate targets based on green technologies require the expansion of electricity networks (D'Amico et al., 2018; Haucap & Pagel, 2016). This is inevitably associated with increased collision risks for several bird species (Erickson et al., 2002). Bird strikes at power lines have been investigated empirically for several decades and have been documented for several hundred species (Bernardino et al., 2018; Loss et al., 2014). However, such collisions can be reduced by adding appropriate markers to the power lines, referred to as “bird flight diverters” (hereafter “diverters”), and numerous studies have investigated several bird species(complexes) in relation to their interplays with different diverter and habitat types (e.g., APLIC, 2012; Barrientos et al., 2011; Liesenjohann et al., 2019). Several studies have demonstrated a reduction in bird strikes due to the diverters, and a recent meta‐analysis estimated a reduction in collisions (averaged over all species, habitats, and diverter types) of approximately 50% (Bernardino et al., 2019).

**FIGURE 1:**
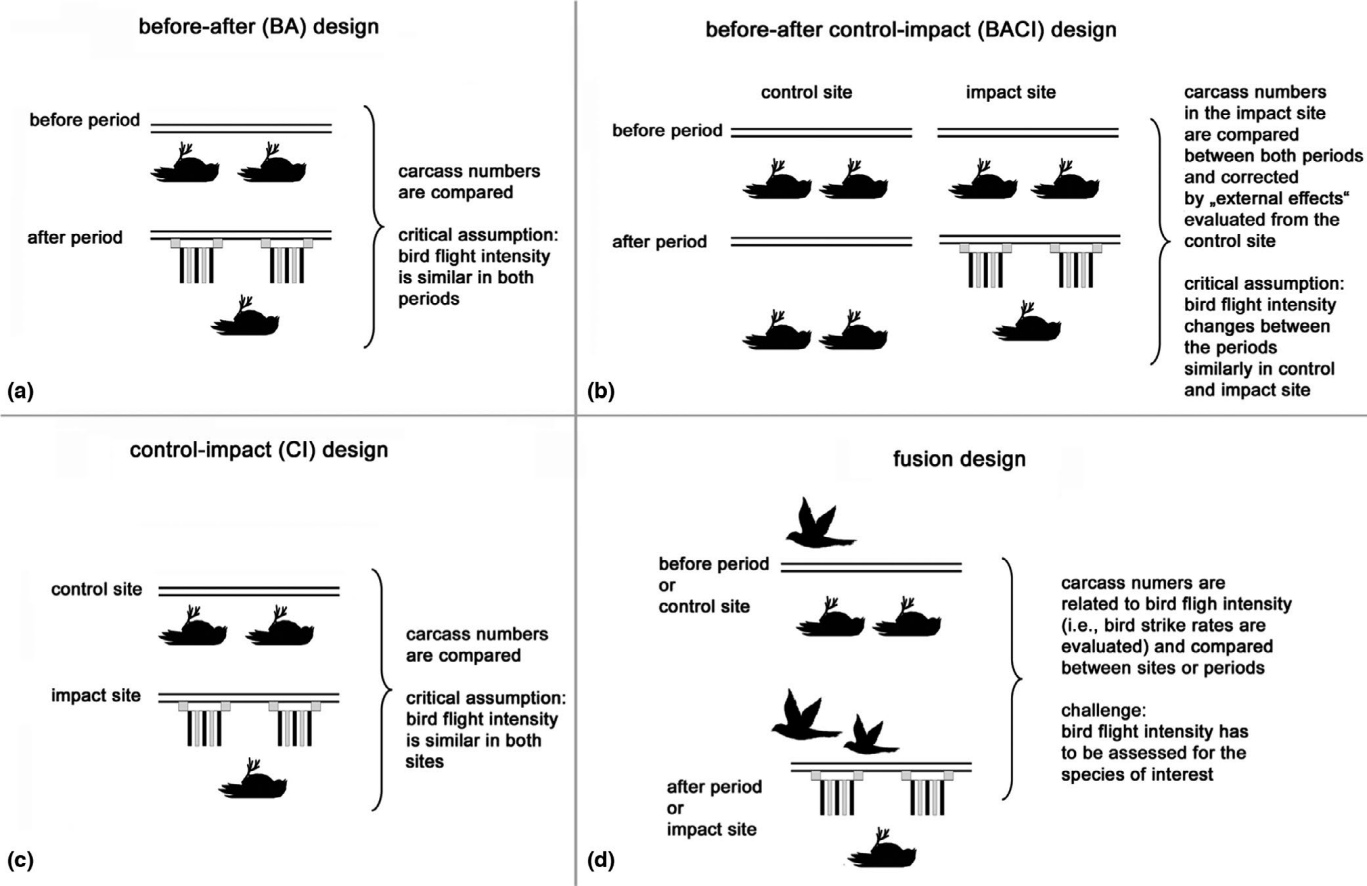
Schematic representation of different field‐experiment designs to evaluate bird flight diverter effectiveness. Before‐after and control‐impact studies (a, c) have previously been used, but have increasingly been replaced by the before‐after control‐impact design (BACI) (b) to reduce bias. However, we demonstrated that even a BACI approach may lead to biased results and propose an alternative fusion model approach (d), in which carcass numbers are directly related to bird flight intensity

Field experiments evaluating diverter effectiveness are usually based on regular carcass searches, comparing numbers between unmarked and marked power lines. In the simplest design, either the same power line is monitored in two subsequent years, with diverters installed prior to the second sampling year (“before‐after (BA) design,” Figure 1a), or two nearby lines (one marked, one unmarked) are compared within the same period (“control‐impact (CI) design,” Figure 1c). However, both these approaches have the potential problem that differences between the obtained carcass numbers may not be exclusively caused by the diverters, but may instead reflect extrinsic fluctuations in flight intensity between the periods (in the BA design) or sites (in the CI design). Such fluctuations could be associated with differences in population size between the two subsequent years, or habitat differences between the two sites. Thus, it has been recently suggested to adjust the (corrected) number of carcasses with flight crossing rates whenever a BA or CI design is adopted (Bernardino et al., 2019). Such correlations between bird flight and strike intensity have been previously considered, for example, in Brown and Drewien (1995), de la Zerda and Evans (2012).

Alternatively, the “before‐after control‐impact (BACI) design” has increasingly been suggested as a means of overcoming this potential bias for impact studies in general (Schwarz, 2014; Smith & Design, 2002) and diverter effectiveness studies in particular (Barrientos et al., 2011, 2012; Bernardino et al., 2019; Jödicke et al., 2018). Using this approach, two sites (an impact and a control site) are monitored in two different periods (before vs. after period), where diverters are only installed for the after period at the impact site. This allows the impact site to be used to determine the preliminary diverter effect, which can subsequently be corrected for extrinsic fluctuations estimated from the control site (c.f., Figure 1c).

However, the BACI design is also subject to the critical assumption that the relative changes in bird flight intensity from one period to the other are similar in both sites (in the following termed “synchronicity assumption”). For example, if the flight intensity in the control site decreased by 50% from the first to the second period, the same relative change is assumed to hold for the control site. If this assumption is not met and the relative change differs between the two sites, the BACI‐based estimated diverter effect will be biased. It is often not known whether this assumption is met, and it may be impossible to prove if regular bird flight activities are not monitored, in addition to carrying out carcass searches.

In this study, we used extensive bird flight data from a bird strike project in Germany (Jödicke et al., 2018) and demonstrated that the synchronicity assumption was frequently violated for different bird species(complexes) and that this violation could in turn lead to strongly biased BACI estimates of diverter effectiveness. To overcome this problem, we seize and elaborate the ideas of Bernardino et al. (2019), Brown and Drewien (1995), de la Zerda and Evans (2012) by presenting an experimental–statistical approach to evaluate bird diverter effectiveness by directly offsetting carcass numbers with observed bird flight intensities (Figure 1d). We call this design “fusion design” (not to be confused with image fusion methods (Han et al., 2016)), since flight and carcass intensities are fused or merged to one single quantity, the latter being proportional to the bird strike rate. On the one hand, using real flight and carcass data for geese (Jödicke et al., 2018) as an example, we compared diverter‐related collision reduction estimates (and certainties) between the BACI and the fusion approaches. On the other hand, based on extensive simulations, we systematically compared the performance of the presented vs. the previous approaches under various data conditions. Further advantages and challenges of the fusion approach are terminally discussed.

## 2 MATERIALS AND METHODS

The analyses presented in this work strongly rely on the flight and carcass data collected in the wire‐marking effectiveness study as presented in Jödicke et al. (2018). The corresponding study area is located near the Elbe River close to Hamburg, Germany. It crosses a sprawling former foreland complex of the Elbmarsch, which was designated as bird sanctuary, that is, parts of the area are conservation respectively bird protection areas. The study area includes nine span fields of a 380‐kV power line with a total length of 4.4 km. The project area was divided into three main sections, two of them mainly including arable land and pasture grassland, where the third one is mainly used as extensive grassland. Impact and control sites were chosen such that both comprise different types of habitats.

### 2.1 Bird flight intensity and synchronicity

We evaluated the frequency with which the synchronicity assumption underlying BACI is met using bird flight data from the largest bird strike study in Germany to date (originally designed as a BACI study from 2016 to 2018, c.f. Jödicke et al., 2018). Here, bird flight movements were recorded weekly within one 300‐m wide corridor on both sides of three different counting points (two within the control site and one within the impact site) with a usual counting duration of 4 hours per count and counting point. All detected birds crossing the lines below, flying through the lines, or crossing them above were recorded. We want to point out that if there are baseline ecological differences between the different counting points, due to the specific type of statistical analyses applied, these effects are subtracted out during BACI analysis respectively the analysis of synchronicity (c.f., below for more details). In each season (before vs. after), 70 counts were carried out. A total of 128 species including >450,000 individuals were observed within 1644 observation hours. More details are given in Jödicke et al. (2018).

For the following analysis, bird count data were assigned to the most common 12 different species(complex) groups to account for differences, for example, in ecology, physiology, and behavior: starlings, waders, geese, ducks, corvids, herons, gulls, doves, swans, cormorants, thrushes, and others. We evaluated the synchronicity assumption by applying an appropriate generalized linear model (GLM; Faraway, 2006; Guisan & Hastie, 2002) to the bird count data, using bird numbers (per counting point‐date‐combination) as the outcome variable, the logarithm of counted hours (per counting point‐date‐combination) as an offset (Korner‐Nievergelt, Roth, et al., 2015), and the expression period+site+period:site as fixed‐effect predictors. In particular, the variable site comprises the two levels “impact site” vs. “control site,” and the variable period comprises the two levels “before period” and “after period,” as defined within a BACI design (Schwarz, 2014; Smith & Design, 2002). The interaction term period:site thus represents a possible violation of the synchronicity assumption (while possible baseline differences are captured by the main effects period and site and thus do not bias the synchronicity analysis), that is, if the corresponding *p*‐value is small, a violation of the assumption is likely. A negative binomial distribution has been assumed to account for (possibly overdispersed) count data (Lindén & Mäntyniemi, 2011). The strength of expected bias in the BACI estimate for the diverter‐related reduction in bird strikes can be calculated directly from the regression coefficient associated with the above interaction term.

### 2.2 Carcass searches and correction

Collision victims were recorded by searching six defined parallel transects of 20 m width (i.e., a total width of 120 m has been searched) below and next to the power line. The total length of the search distance was 4.1 km, resulting in a total transect length of 24.6 km per carcass search. Searches were carried out every 2 days during the main migration and resting periods (February 15 to April 30 and August 15 to October 31) and every 5 days at other times. A total of 222 carcass searches were carried out over two complete seasons (from February 2014 to April 2016). A total of 637 collision victims were found.

The number of found carcasses usually underestimates the true number, because (1) not all collided animals die within the search area, (2) not all carcasses persist until the next search (due to decomposition and removal by scavengers), and (3) not all remaining carcasses are detected by the searchers (Korner‐Nievergelt, Behr, et al., 2015). Additional complexity is introduced by the fact that carcasses may be overlooked in one search but subsequently found during a following search (Korner‐Nievergelt, Behr, et al., 2015). Various additional field experiments have been carried out and analyzed to inform the corresponding correction factors, as detailed in Jödicke et al. (2018), Mercker (2021). In particular, correction factors were calculated separately for each carcass based on the regression results from these additional experimental data. Various covariates (e.g., vegetation parameters, carcass size, or the number of days to the previous search) were included in the calculation. The application of these corrections led to an estimated total of 1224 carcasses, in average the correction factor was 1.9, ranging from 1.1 to 10.0.

### 2.3 BACI and BA analyses

We conducted BACI analyses using appropriate modern regression methods (McDonald et al., 2000; Schwarz, 2014; Smith & Design, 2002) allowing for flexible adaptation to several challenges associated with ecological data, including autocorrelation or overdispersed count data (Mendel et al., 2019; Mercker, 2021; Schwarz, 2014). Notably, we used the (corrected) number of carcasses (per search‐date‐site combination) as the outcome variable in a GLM and prescribed a negative binomial distribution to account for (possibly overdispersed) count data (Lindén & Mäntyniemi, 2011). As fixed‐effect predictors, the expression period+site+period:site has been used, where the variable site comprises the two levels “impact site” vs. “control site,” and the variable period comprises the levels “before period” and “after period.” If the nonsimulated (corrected) caracass data from Jödicke et al. (2018) were used, the additional variable Jday (Julian day) was included as a fixed‐effect predictor, to correct for seasonal effects. In particular, the variable was introduced as a cyclic regression spline allowing for both nonlinear dependencies and the cyclic nature of this dependency on the other (Wood, 2017). A generalized additive model (GAM; Hastie & Tibshirani, 1990; Wood, 2017) was thus fitted to the data. All underlying model assumptions (including possible temporal autocorrelation) were checked. In the case of the simulation study (c.f., below), dependency on the Julian day was not considered for the sake of complexity and seasonal effects were not simulated. We want to point out that for the sake of complexity, in all BACI, BA, and fusion analyses (for the latter c.f., below), we treated the corrected carcass data as if they were sharp data. We think this is justifiable as this is primarily a comparative study. If the focus were instead on the total strength of diverter effects (and their confidence intervals), one would have to propagate the uncertainties from the correction factors into final regression standard errors. This issue is frequently neglected in the analysis of corrected bird strike data. Appropriate methods for propagation include, for example, bootstrapping/resampling techniques (Mercker, 2021).

For the sake of completeness, we included a BA model (mathematically equivalent to the CI approach—c.f., Figure 1) in the comparative simulation study. This model was similar to the above‐described BACI regression model, but was only applied to the data for the control site, and period was used as a fixed‐effect predictor instead of period+site+period:site.

### 2.4 Fusion analysis

For the fusion analysis, carcass data were restricted to the impact site and were temporally correlated with observed bird flight data. For the latter, all flight observation data for the period between two carcass searches were pooled (separately for each analyzed species(complex)) and attributed to the carcass numbers for the second search. If there were no flight data within this period, the subsequent carcass search was used instead. The variable Jday was only used if the models were applied to the nonsimulated flight and carcass data from Jödicke et al. (2018). For the statistical fusion analysis, we developed and compared two different possible approaches.

#### 2.4.1 Negative binomial (NB) fusion model

For the “NB fusion model,” we first calculated the measure A=days×individuals/hours from the temporally pooled bird flight data (c.f., above), where days depicts the (possibly varying) number of days for which the bird flight data have been pooled (i.e., the number of days between the corresponding carcass searches), individuals is the count of flying birds, and hours measures the total duration of flight observations in the considered period. This measure can be used in the GAM as an offset by

$$\log\left(N\_carcass_{i}\right)=b+s\left(Jday_{i}\right)+Period_{i}+offset\left(\log\left(1+A_{i}\right)\right)+\varepsilon_{i},$$

where i is the index for the observation, $N\_carcass_{i}$ depicts the (corrected) carcass number, $s\left(.\right)$ is a cyclic regression spline, and for the model residual, it holds that $\varepsilon_{i}:N\left(0,\sigma \right)$ is independent and identically distributed. Mathematical reformulation based on the properties of the log‐link function reveals that the expression $\left(N\_carcass/day\right)$ is related to/divided by the expression $\left(individuals/hours\right)$, and bird strike intensity is thus related to flight intensity.

#### 2.4.2 Binomial (B) fusion model

Alternatively, a binomial GLM/GAM can be applied to the data (“B fusion model”) to realize the fusion framework. Within such a binomial model, the response variable consists of two integer non‐negative numbers, counting the number of events (in this case, carcasses per day) and the number of nonevents (in this case, observed bird crossings per hour) for each observation and relating them to each other. We thus defined $y_{1}=round\left(N\_carcass/day\right)$ and $y_{2}=individuals/hours$ and considered

$$\text{logit}\left(y_{1},y_{2}\right)=b+s\left(\mathrm{Jday}\right)+Period+\varepsilon_{i}.$$

Because only integer numbers can be used in the outcome variable of binomial models, $y_{1}$ and $y_{2}$ depict rounded numbers, possibly introducing additional bias. However, this can be minimized by multiplying N_carcass/day and individuals/hours with the same large constant c (e.g., c=10,000) before rounding.

### 2.5 Simulation study

We compared the performances of the fusion models with the BACI and BA approaches based on a simulation study and evaluated relative bias, statistical power (related to type II errors), and false‐positive rates (type I errors). Since the geese complex comprised the largest share of the data in Jödicke et al. (2018); 61% of total number of counted birds, we used these data as a basis—in terms of general data structure and density, and also with respect to the mean values and variances of bird flight activity and carcass numbers/bird strike rates. Thus, we ensure that the simulation study was based on a realistic scenario.

In the performed simulation study, we can distinguish between two separate Monte Carlo‐based simulation processes. On one hand, based on the means and variances extracted from the real geese data, we simulated the stochasticity of bird flight numbers and resulting collision victims. Here, a random negative binomial distribution was assumed for bird flight numbers (separately simulated for each date‐site combination), and a binomial distribution to simulate (separately for each single crossed bird) a possible collision event. Related parameters are given in lower case Greek letters. On the other hand, a set of parameters representing flight‐intensity differences between sites and/or periods, as well as the strength of diverter‐induced collision risk, need to be set prior to each simulated dataset. These parameters were chosen randomly from a uniform distribution and are depicted by capital Greek letters.

Based on these two stochastic processes, we simulated 20,000 different datasets and evaluated the performances of the BACI, BA, NB fusion, and B fusion approaches. In particular, in 10,000 of these datasets, we set the diverter‐induced reduction strength to zero to evaluate the false‐positive rate. In the other half, the reduction strength is set to values >0 (c.f., below) to analyze the statistical power. Furthermore, a site‐spanning difference in flight intensity between the periods and a violation of the synchronicity assumption were both possible.

The statistical power and false‐positive rates were evaluated by applying appropriate GAMs. Here, the binomial variable “significant effect” vs. “no significant effect” was used as the outcome variable, with variables possibly influencing these rates as predictors. Notably, the method variable (BA vs. BACI vs. NB fusion vs. B fusion) was used as a fixed effect, and further variables describing differences in flight intensities between periods and/or sites (such as a variable measuring the violation of synchronicity) or the underlying diverter‐induced reduction strength (c.f., below) were formulated within a tensor product spline (Wood, 2017) allowing arbitrary nonlinear dependencies and interactions.

For the Monte Carlo simulations of bird/victim numbers, we extracted the negative binomial parameters $\mu_{1}=1,254$ and $\theta_{1}=0.41$ for (pooled) flight observations. The collision rate was calculated as ζ=0.001, which is not the real rate (e.g., in terms of collisions per number of total crossings), but rather the number of carcasses relative to the number of *observed* crossings. For each simulated bird separately, we used ζ as the probability in a random binomial distribution to simulate the event of a bird strike.

With respect to the prescribed differences in flight intensities, we randomly chose $\Omega \in \left[1.0,2.5\right]$ to represent the average increase in flight intensity from the before to the after period (in both sites). Further, $\Gamma \in \left[1.0,2.5\right]$ represents the strength of bias with respect to synchronicity. Finally, the reduction in strength of collision probability is represented by $\Lambda \in \left[0.1,0.8\right]$, for example, a value of Λ=0.3 means that the collision risk α is reduced by 30% in the after period and impact site. If diverter‐induced reduction effects exist (i.e., Λ>0), the insensitivity of the BA/CI and BACI approaches to differences in flight intensity might either counteract these effects (i.e., reducing the statistical power), or lead to overestimates of diverter effectiveness, depending on the direction of bias induced by differences in flight intensity. To highlight this, in the simulation study, we chose Γ such that it counteracted the diverter‐induced reduction effect in the BA and BACI models. Finally, for all GAM‐based evaluations of model performances (Figure 3a–d,f), if not otherwise stated, Ω = 1.0, Γ = 1.0, and Λ = 0.5 was assumed, that is, only the partial effect of each specific variable was analyzed, preventing cross‐interactions with the other variables.

### 2.6 Software and packages

All statistical analyses were carried out using the open‐source software R version 4.1.0 (R Core Team, 2016). The package *mgcv* (Wood, 2017) was used for all GLM and GAM analyses, and *ggplot2* (Wickham, 2009) was used for visualization.

## 3 RESULTS

### 3.1 Assumption of synchronicity

**FIGURE 2:**
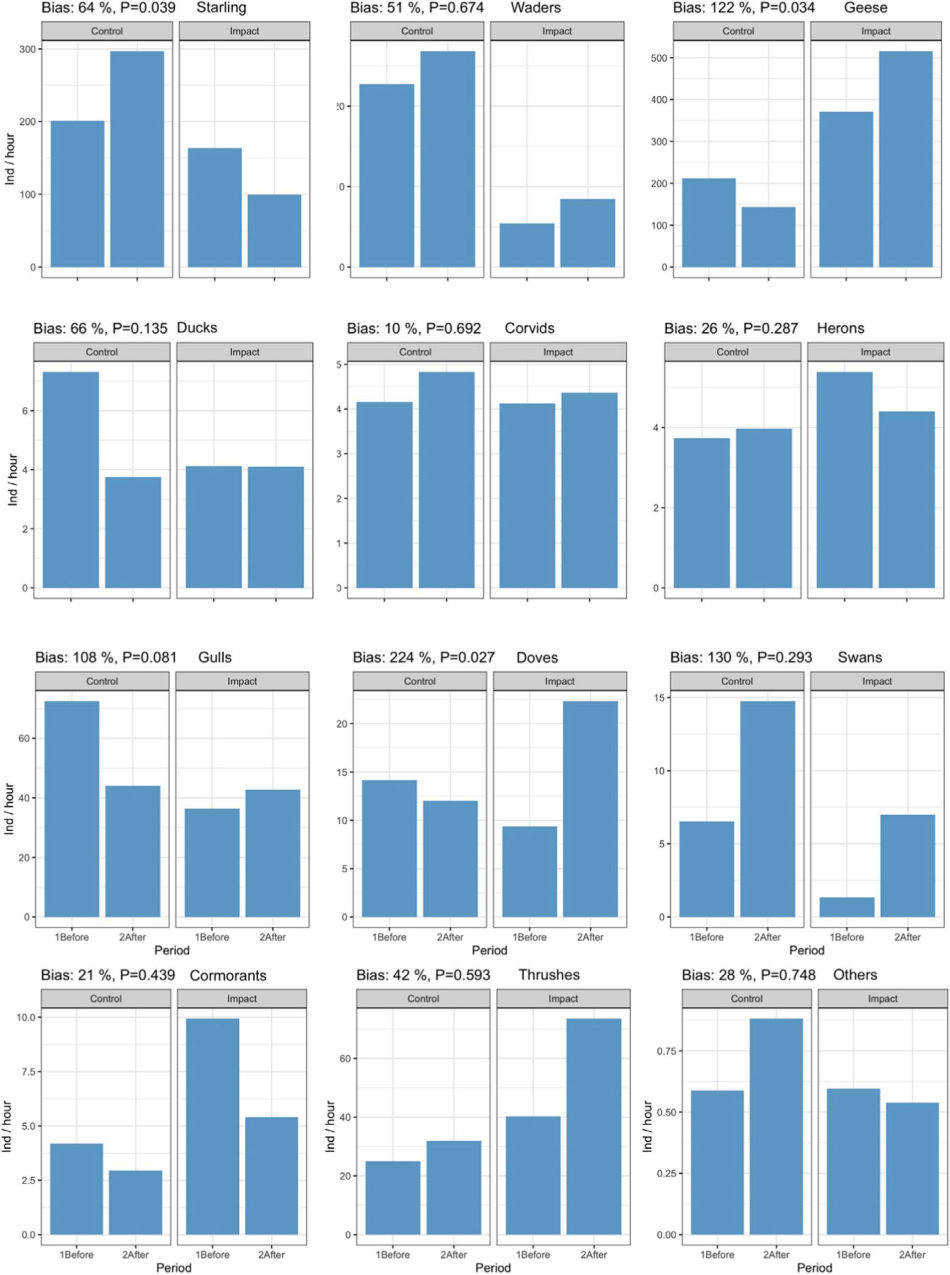
Bird flight intensity at power lines in the control vs. impact sites, separately evaluated for the before vs. after periods. For 4 of the 12 analyzed bird species(complexes), the statistical analyses indicates a violation of the BACI assumption of synchronicity (*p* < .1) and consequently biased BACI estimates (c.f., header of subfigures for estimates of bias strength and significance)

The GLM analysis of the bird count data revealed that the predicted deviation from synchronicity (and thus expected bias of the estimated BACI effect of diverter effectiveness) for 7 out of 12 bird species(complexes) was >50%, with an average (over all 12 species(complexes)) of 74% (Figure 2). For 4 of these species(complexes), namely starlings, geese, gulls, and doves, the violation was significant to the level of α=0.1. These 4 groups comprised the majority (69.7%) of the >450,000 counted individuals, and the expected BACI‐bias ranged from 64% to 224%.

Graphical analyses (Figure 2) for different species(complexes) demonstrated that the interannual increase at the impact site was stronger than that at the control site (c.f., e.g., geese, gulls, and doves in Figure 2), indicating that the observed violation of synchronicity was not caused by a scaring effect of the diverters, resulting in displacement to other sites. This is in accord with the assumption that diverters cause reactions at relatively short distances but have no large‐scale scaring effects. Furthermore, the qualitative and quantitative heterogeneity in the observed differences in flight intensity between sites and/or periods (c.f., e.g., opposite relative changes of starlings vs. geese between the periods—Figure 2) suggests that reasons for these violations were highly species(complex)‐specific and were thus unlikely to be due to a single (rare) study‐specific event, such as regular disturbances in the control site and after period.

We thus conclude that violation of synchronicity is not a rare event, but may occur frequently, probably with species(complex)‐specific causes. These violations were generally relatively strong and may thus result in corresponding bias in BACI‐based estimates of diverter effectiveness, with an estimated mean bias of well over 70%. Possible reasons for these fluctuations include changes in the size of local populations, changes in land use (within years or even within a season—directly on site and also in the surrounding area (transfer flights)), and finally the weather, which is assumed to have a strong influence on local land use by birds, including small‐scale effects (e.g., when areas are nonregularly covered by water) and larger scale effects (e.g., when lakes freeze over).

### 3.2 Effectiveness estimates from geese data: BACI vs. fusion approaches

The geese complex comprised the largest share of the data in Jödicke et al. (2018), and we therefore used this species complex to compare the BACI‐based results with those of the proposed fusion approach. An overview table with respect to the total number of geese carcasses, flight observations, and hours of conducted flight surveys is given in Table 1. The BACI approach gave an estimated reduction strength of 39.1% (95% confidence interval: [−65.3%, 77.6%], *p* = .33), the NB fusion model estimated a reduction of 89.2% (95% confidence interval: [75.6%, 95.2%], *p *< .0001), and the B fusion model estimated a reduction of 92.8% (95%‐confidence interval: [76.1%, 97.8%], *p *< .0001). Here and in the following, “reduction” abbreviates “diverter‐induced collision‐reduction.”

**TABLE 1:** Total number of (corrected) geese carcasses, flight observations, and hours of flight surveys in the different sites and periods

Period	Site	Sum_carcass	Sum_flight	Hours
Before	Impact	164.01	76,890	218.83
Before	Control	69.67	76,554	493.75
After	Impact	51.49	115,043	270.25
After	Control	36.34	48,670	405.75

Both fusion approaches thus produced similar estimated effect sizes, which were more than twice that of the BACI estimate, in accordance with a BACI bias of 122% estimated by synchronicity analysis (Figure 2). This suggests that the BACI‐based estimate was strongly underestimated. Furthermore, the corresponding precisions of the fusion estimates (e.g., confidence intervals and *p*‐values) were better than those of the BACI method. Finally, the BACI‐GAM showed temporal autocorrelation within the model residuals, which had to be included as an appropriate autocorrelation structure. The underlying cause was a distinct dependency of carcass numbers on time, induced by the seasonality of bird flight activity, driven by, for example, feeding, resting, or migration periods. In contrast, the fusion models showed no temporal autocorrelation, because carcass numbers were directly related to bird flight intensities, and corresponding seasonal effects were thus canceled out. Temporal dependencies may only occur if bird strikes *rates* show seasonality, for example, if migrating birds show an increased collision risk compared with breeding birds. This may however occur due to habituation effects playing a role for birds that remain on site for a longer period (e.g., wintering and/or breeding season). The lack of temporal autocorrelation in the fusion models thus facilitates the statistical analysis compared with BACI.

### 3.3 Comparative simulation analysis

**FIGURE 3:**
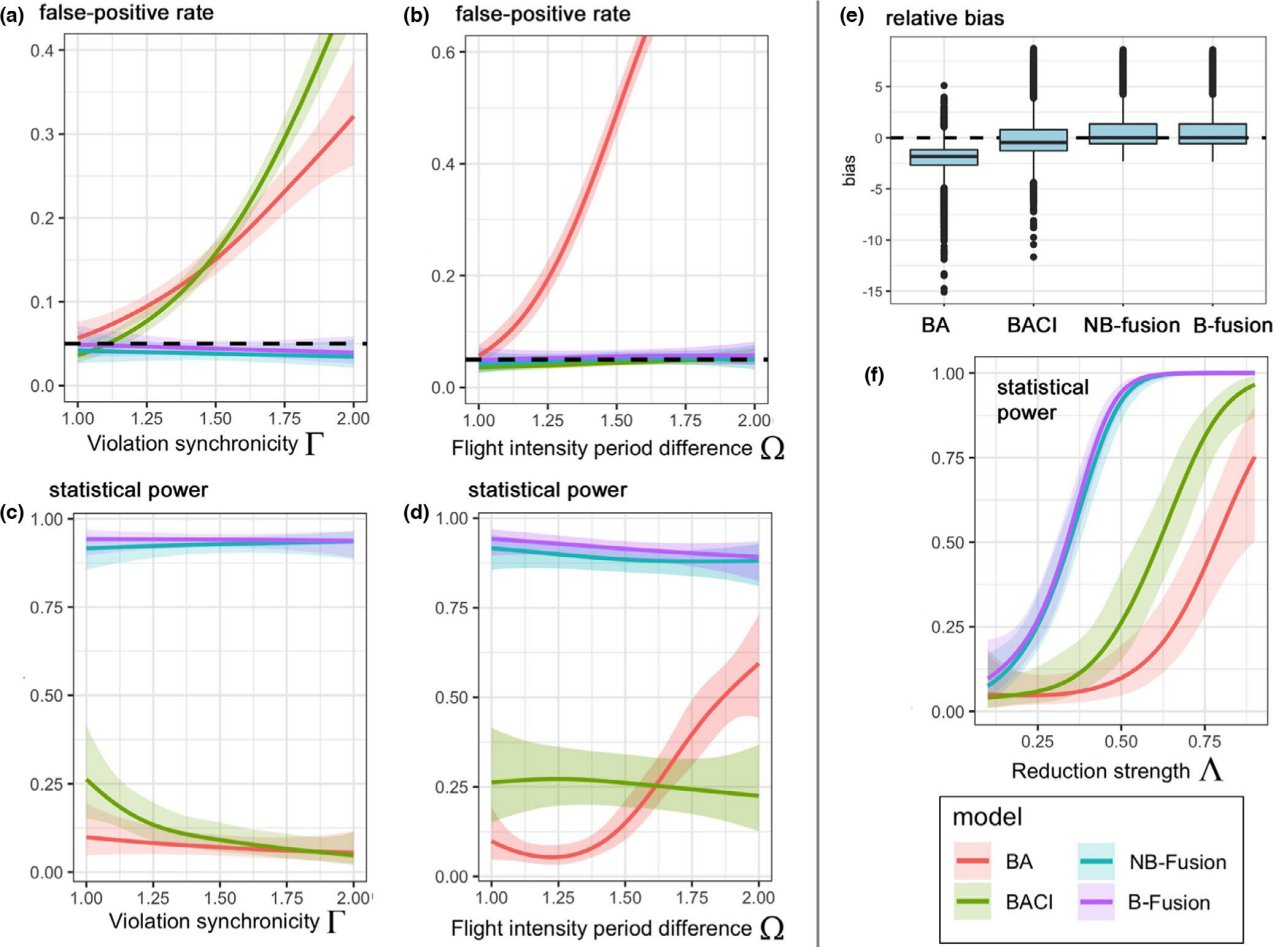
Simulation‐based comparative performances of BA (before‐after), BACI (before‐after control‐impact), NB (negative binomial) fusion, and B (binomial) fusion approaches for evaluating diverter effectiveness. Models were compared in terms of false‐positive rates (a, b), statistical power (c, d, f) and relative bias (e). (a–d, f) GAM‐based results, (e) boxplot analysis. In (a–d, f), shaded areas depict 95% confidence bands. For all GAM‐based analyses, Ω = 1.0, Γ = 1.0, and Λ = 0.5 is assumed, if not otherwise stated (c.f., x‐axes). In (a, b), black dashed lines indicate nominal false‐positive level of *α* =.05; in (e) dashed line represents zero bias

The results of the comparative simulation analysis are shown in Figure 3. With respect to bias, as expected, the BA approach generally showed the strongest negative bias (Figure 3e), given that both differences in flight intensity between the periods (Ω) and the violation of synchronicity (Γ) induced bias in these estimates. This was followed by the BACI method, which was only biased if the synchronicity assumption was violated. In contrast to the BA and BACI approaches, both fusion approaches generally produced unbiased results (Figure 3e), as a result of the direct relationship between bird strike victims and flight intensity.

The analysis of false‐positive rates (Figure 3a,b) showed a constant nominal level of α= .05 for both fusion approaches, whereas the false‐positive rate for the BA approach increased strongly with both differences in flight intensity between the periods (Ω) and the violation of synchronicity (Γ). The latter also holds for the BACI approach, where, for example, a violation of synchronicity of 74% (average for the real geese data—c.f., above) led to a false‐positive rate of approximately 0.3, that is, a 6‐fold increase compared with the nominal level. Thus, if the average violation of synchronicity (estimated from Jödicke et al., 2018) is representative, there is a 1/3 chance of obtaining false‐significant results in a BACI study, where no reduction effect exists. In the case of a BA (or analogously CI) design, the situation is even worse, because the effect of different flight intensities between the periods also contributes to the false‐positive rate. Here, the combination of both effects may lead to real false‐positive rates greater than 0.5 instead of 0.05, that is, 50% of the significant effects are false‐positives, in scenarios where no reduction effect actually exists.

Figure 3f shows the statistical power of the different approaches depending on the diverter‐induced reduction effect (Λ). As expected, the power increased with increasing Λ for all methods, that is, the stronger the diverter‐induced reduction, the more likely that a significant effect would be detected. However, there were distinct quantitative differences in the powers of the methods: the BA approach showed the lowest power, followed by the BACI approach, while both fusion approaches showed the highest statistical powers for all reduction‐strength values Λ. This result is striking, given that there was no bias in this plot/analysis (i.e., Ω=Γ=1.0 prescribed for the GAM‐based predictions, considering the scenario where flight intensity is homogeneous across all periods and sites). Thus, even under a completely unbiased scenario (i.e., with respect to BACI, no violation of synchronicity), the fusion approaches had distinctly higher powers. After integrating the overall reduction‐strength values $\Lambda \in \left[0.1,0.9\right]$, the power of the fusion approaches in the unbiased scenario was approximately three times higher compared with the BACI approach.

The difference between the BA/BACI and fusion approaches was even stronger in biased scenarios (probably most real cases, as demonstrated in this study): With increasing violation of synchronicity Γ (assuming Ω=1.0 and Λ=0.5), the statistical power of the BACI and especially the BA approach reduced to zero, whereas the fusion approaches showed a nearly constant power of approx. 0.9. (Figure 3c). If the difference in flight intensity between the period, Ω, increased (assuming Γ=1.0 and Λ=0.5), the powers of the BACI and both fusion approaches decreased slightly, whereas the BA approach showed a distinct increase in power with Ω. However, the latter was an artifact of the false‐positive rate, which increased strongly with this variable for the BA approach (Figure 3b), also affecting simulations where a reduction effect existed.

The present comparative results could however depend strongly on the specific flight intensities and variances and/or bird strike rates as extracted from the data in Jödicke et al. (2018), and the performances of the different methods could be distinctly different for other values/scenarios, implying limited generalizability of our results. However, this is most likely not the case, given that both changes in flight intensity and in bird strike rates impact directly on carcass numbers, the latter representing the bottleneck of data density in all investigated approaches.

To further demonstrate this, we repeated the simulation study twice considering the following scenarios: (1) a strongly reduced average flight intensity (by a factor of 100, but increasing bird strike rates by the same factor to obtain an average number of carcasses distinctly above zero) and (2) a strongly increased variance/spread in flight numbers by changing $\theta_{1}$ from 0.41 to 0.2. The fusion models still showed an average increase in statistical power compared with the BACI approach of 2.44 times (in unbiased scenarios) in scenario (1) (c.f., Figure S1), and 4.98 times in scenario (2) (c.f., Figure S2). The latter can be explained by the fact that a higher stochasticity in flight numbers also caused a corresponding spread of carcass numbers, which seemed to negatively affect the BACI approach more than the fusion methods. Finally, the applied simulation approach deviated from the real scenario in that all birds potentially colliding with the power lines were observed. To exclude a corresponding bias of the presented results, we repeated the simulation generating two different random bird flight numbers: a negative binomial‐distributed number underlying the virtual bird strike events and a random binomial distribution with probability of *p* = .01 for the observed birds (forwarded to the model, i.e., in this scenario only 1% of the crossing birds were observed). The results confirmed an average 2.95 times higher statistical power for the fusion models, which thus appeared to be insensitive to these changes.

Our results revealed only minor performance differences between the NB and B fusion approaches. Both models showed nearly indistinguishable results in terms of estimated bias, false‐positive rates, and statistical power (Figure 3). Based on our practical experience however, we observed that the NB approach showed better convergence properties (particularly if data are sparse). Furthermore, the need for rounded numbers in the B approach may introduce bias. Finally, the calculation of reduction strength is more straightforward in the NB framework, especially if several predictors are involved. Taken together, we considered that the NB fusion approach was preferable to the B fusion‐based evaluation.

## 4 CONCLUSIONS AND OUTLOOK

Using extensive bird flight data from the largest bird strike study in Germany to date, we demonstrated that a critical assumption underlying BACI analysis, that is, the assumption of synchronicity, was frequently and distinctly violated for different bird species (complexes). This may result in distinct bias when estimating reductions in bird strikes due to the use of flight diverters using a BACI approach. We present an alternative field‐ and analysis‐based design in which bird strike victims are offset with bird flight intensity data close to the power lines (“fusion approach”). Taking geese flight and carcass numbers from Jödicke et al. (2018) as an example, we demonstrated that the BACI approach estimated a diverter‐induced reduction effect (nonsignificant) of approximately 40%, whereas the fusion models estimated highly significant effects of approximately 90%. This difference was in accordance with the estimated BACI bias based on flight intensity data alone (by estimating the strength of violation of the synchronicity assumption).

We further systematically compared the BACI approach with the presented fusion methods by carrying out an extensive simulation study to investigate bias, false‐positive rates, and statistical power. We demonstrated that BACI (and even more BA and CI approaches) could exceed the nominal false‐positive rates in practice, in contrast to the fusion approaches that showed nominal type I error rates. Furthermore, even under completely unbiased scenarios, the fusion models had an average 3 times higher statistical power compared with BACI, with comparable experimental effort.

Importantly, the field‐experiment efforts required by the BACI and fusion approaches can be comparable: For BACI, twice the length of the power line has to be monitored (because impact and control sites are required), whereas for the fusion approach, bird flight has to be monitored in addition to carcass searches. In the case of the study underlying the present results and simulations (Jödicke et al., 2018), we estimated the experimental costs of a pure BACI design to be approximately 1.5 times higher than that for the fusion design, because carcass searches require more manpower and are conducted more frequently than flight observations. However, we want to point out that in the project of Jödicke et al. (2018), the high frequency of searches might be uncommon, limiting the comparability to other studies where less frequent searches are a more likely situation—mainly due to frequent logistical and financial limitations in wire‐marking effectiveness studies. Additionally, the cost ratio may be reversed in regions where flight intensity is lower and bird activity is more evenly distributed during the daytime, requiring more extensive flight observations (Arno Reinhardt, Frank Bernshausen, personal communication). Furthermore, flight observations require highly skilled ornithologists in contrast to carcass searching, which requires less expertise, potentially increasing the costs of the fusion approach. Finally, the BACI design may take advantage of data from previous carcass searches and thus may strongly reduce the additional survey effort if such data exist.

Furthermore, the fusion approach can be used to monitor either one site in two periods (marked vs. unmarked period) or two sites in one period (marked vs. unmarked site), which are mathematically equivalent. In the latter case, the fieldwork can be completed within a single season. However, in this case, it is important that the comparability of both sites is thoroughly checked. Although differences in bird‐flight intensity between the sites are canceled out in the context of the fusion design, other habitat differences that will result in differences in species composition and behavior (such as power lines spanning water bodies, resting areas below the lines, or regular disturbance of resting birds) may distinctly limit the comparability.

If the fusion design is applied, regular bird observations offer the possibility for direct analysis of bird reactions to unmarked and marked power lines, as well as possible collisions. Together with additional covariates (e.g., weather, habitat, power line‐ or diverter‐specific information, and/or disturbances), this may help us to understand the mechanisms of bird strikes. Furthermore, it also offers the possibility of calculating not only relative differences between bird strike probabilities, but also estimating bird strike *rates* (e.g., collided birds per 1000 crossings). However, this requires the total number of crossings to be estimated, which is only possible if regular bird flight observations are sufficiently varied with respect to time and location, that is, an appropriate sampling design is chosen.

A challenge of the fusion method (compared with carcass‐based approaches without additional flight monitoring) is that only bird species that fly mainly during the daytime can be evaluated using standard observer‐based bird counts. However, unbiased estimates may also be obtained if individuals of a species(complex) fly mainly at night, as long as their day‐flight intensity is assumed to change proportionally with the level of night‐flight activity (i.e., days with low flight intensities indicate lower intensities at night and vice versa). Monitoring flight intensity is more challenging for species(complexes) that fly exclusively at night (such as night migrants or several owl species during foraging). Here, nocturnal surveys using night vision scopes (e.g., Murphy et al., 2016) are a possibility, but also recent developments using radar monitoring methods (e.g., Hartman et al., 2010; Pavon‐Jordan et al., 2020) or machine‐learning analyses of audio signals (e.g., Acevedo et al., 2009) may soon help to overcome this limitation. Finally, for applying the fusion design, a certain minimum frequency and length of flight surveys has to be conducted, otherwise the statistical power of the analysis is expected to decrease due to the nonrobustness of flight intensity estimates. In Jödicke et al. (2018), the ratio of flight vs. carcass surveys was approximately 1:1.6, with an average length of 4 hours per count and counting point. The optimal ratio may however strongly depend on local and/or species‐specific conditions and research questions.

In summary, the distinctly higher power compared to the BACI design, in addition to the lower false‐positive rates and the fact that the results remain unbiased even if synchronicity is violated, makes the fusion approach an attractive tool for evaluating diverter efficiency. Such sensitive and unbiased methods are especially important against a background in which species‐, habitat‐, and diverter‐specific reduction strengths are increasingly moving into scientific and political focus. The fusion approach has the added advantages of providing further insights into bird reactions/collisions, estimation of total collision rates, and the possibility of conducting fieldwork within a single season. This approach may thus provide a new standard for evaluating diverter effectiveness, leading to a more precise and comprehensive picture of bird strikes at power lines and how they may be prevented.

## CONFLICT OF INTEREST

The authors declare that they have no known competing financial interests or personal relationships that could have appeared to influence the work reported in this paper.

## AUTHOR CONTRIBUTIONS

**Moritz Mercker:** Conceptualization (lead); Formal analysis (lead); Investigation (equal); Methodology (lead); Visualization (lead); Writing‐original draft (lead); Writing‐review and editing (equal). **Klaus Jödicke:** Conceptualization (supporting); Methodology (supporting); Project administration (lead); Writing‐review and editing (equal).

## Supporting information

Fig S1

Fig S2

## Data Availability

Flight intensity data for starling, geese gulls, and doves (figure 2—data from Ref. Jödicke et al., 2018) as well as geese flight and carcass data from Jödicke et al. (2018) (e.g., underlying the simulated data) are provided on the publicly accessible repository Dryad (https://doi.org/10.5061/dryad.1rn8pk0vj).
