# Supplementary figures and images for: Beyond BACI: Offsetting carcass numbers with flight intensity to improve risk assessments of bird collisions with power lines
Source: Ecol Evol. 2021 Nov 10;11(23):16716–26. doi: 10.1002/ece3.8291 (PMC8668741; doi:10.1002/ece3.8291)

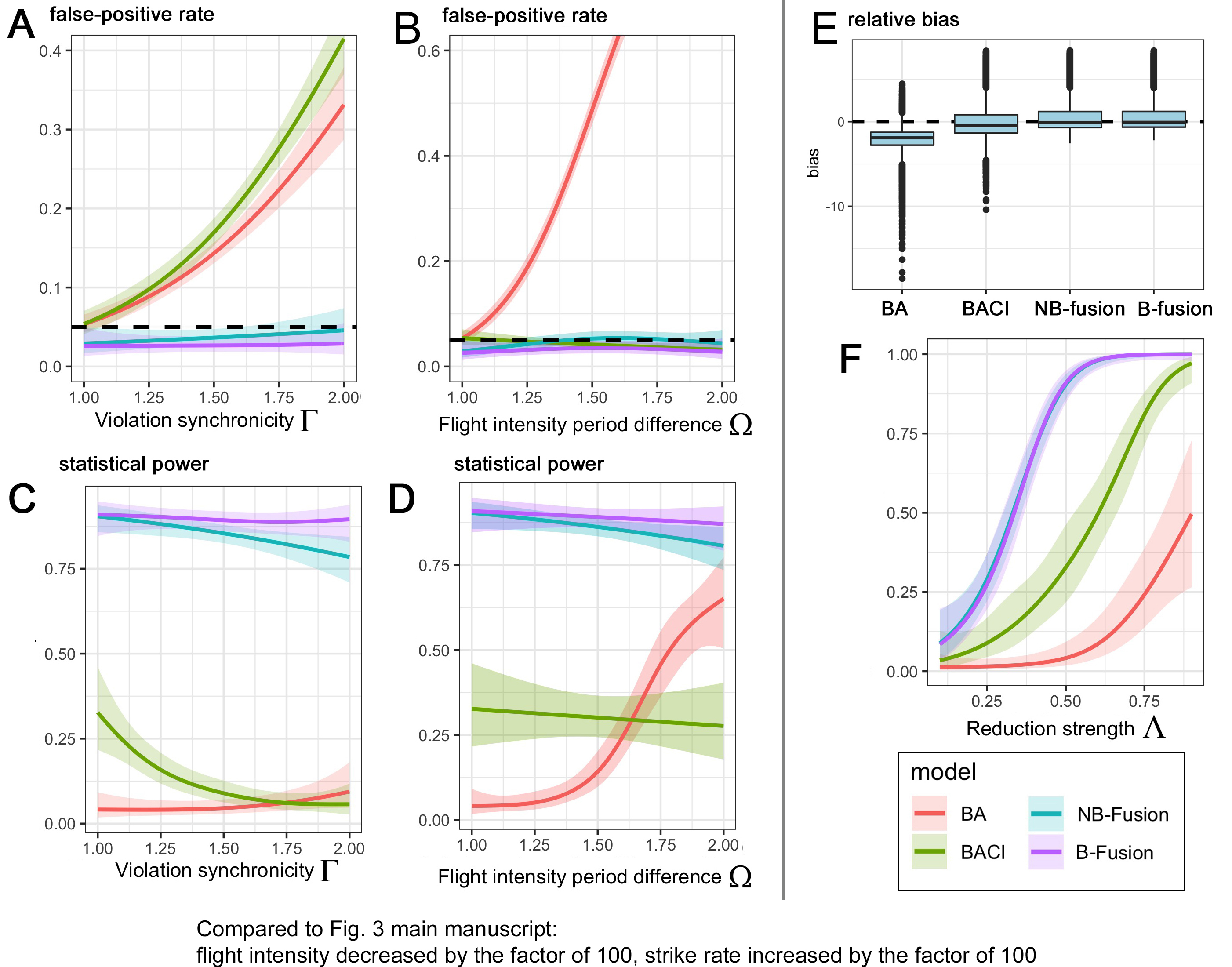

Supplement: Supplementary file 1 — Fig S1 [file ECE3-11-16716-s002.jpg]

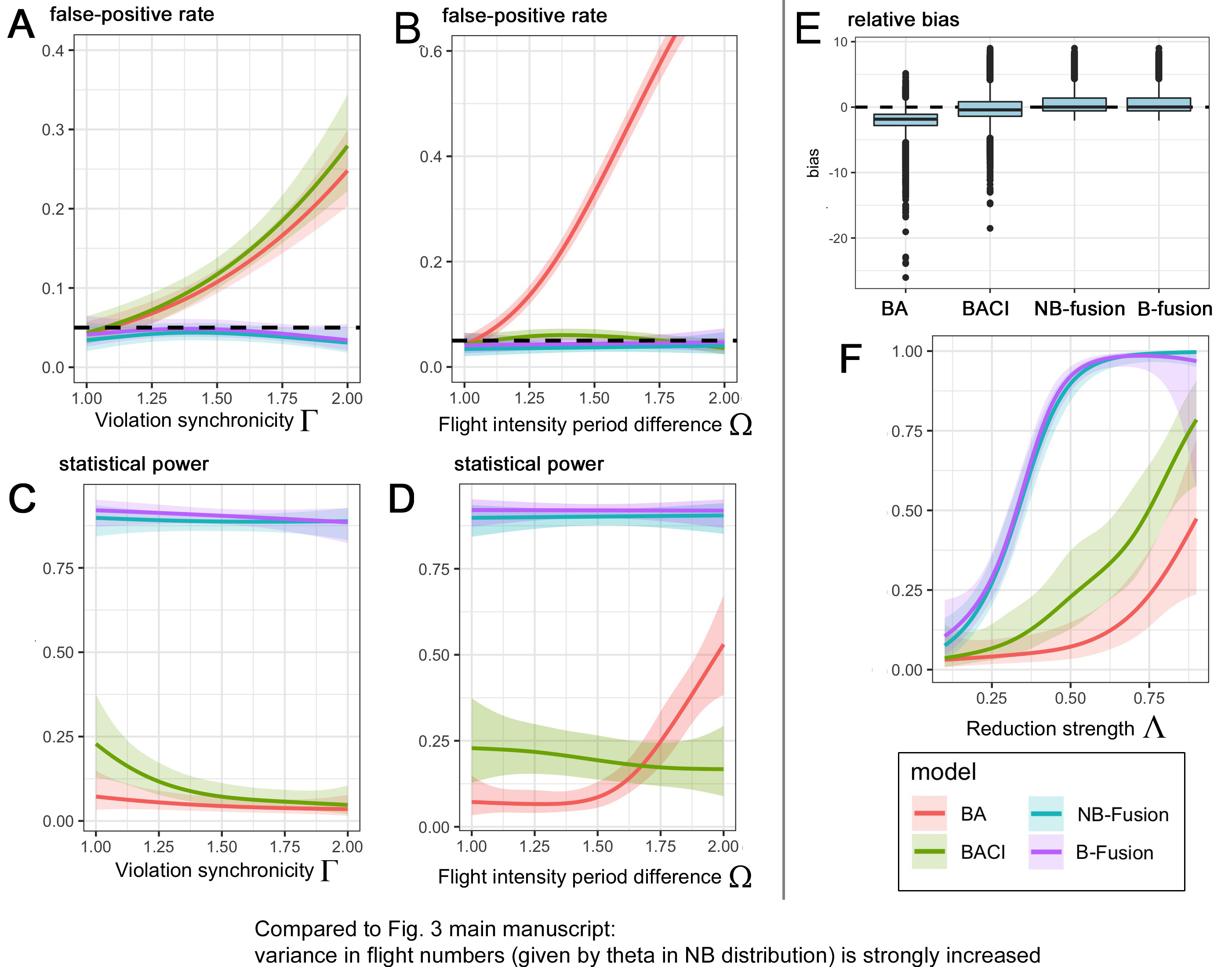

Supplement: Supplementary file 2 — Fig S2 [file ECE3-11-16716-s001.jpg]
